# COVID-19 in Cyanotic Congenital Heart Disease

**DOI:** 10.1155/2023/5561159

**Published:** 2023-04-18

**Authors:** Lama A Ammar, Joseph E Nassar, Fadi Bitar, Mariam Arabi

**Affiliations:** Faculty of Medicine and Medical Center, American University of Beirut, Bliss Street, 11-0236, Riad El-Solh, Beirut 1107-2020, Lebanon

## Abstract

Congenital heart disease (CHD) is the most prevalent congenital defect in newborn infants. Due to the various types of heart abnormalities, CHD can have a wide range of symptoms. Cardiac lesions comprise a range of different types and accordingly varying severities. It is highly helpful to classify CHD into cyanotic and acyanotic heart diseases. In this review, we are investigating the course of Coronavirus disease 2019 (COVID-19) in cyanotic CHD patients. The infection may directly or indirectly affect the heart by affecting the respiratory system and other organs. The effect on the heart that is pressure- or volume-overloaded in the context of CHD is theoretically more severe. Patients with CHD are at a higher risk of mortality from COVID-19 infection or suffering worse complications. While the anatomic complexity of CHD does not seem to predict the severity of infection, patients with worse physiological stages are more susceptible such as cyanosis and pulmonary hypertension. Patients with CHD exhibit continuous hypoxemia and have lower oxygen saturations because of a right-to-left shunt. Such individuals run the danger of rapidly deteriorating in the event of respiratory tract infections with inadequate oxygenation. Additionally, these patients have a higher risk of paradoxical embolism. Hence, critical care should be given to cyanotic heart disease patients with COVID-19 in comparison to acyanotic patients and this is through proper management, close observation, and adequate medical therapy.

## 1. Congenital Heart Disease

### 1.1. General Overview

CHD is the most prevalent congenital defect in newborn infants [1]. CHD accounts for about 30% of all congenital abnormalities. Its prevalence varies significantly amongst populations, averaging 8 per 1,000 live births per year. Due to advancements in screening and detecting techniques, the incidence of CHD has increased in recent decades [2].

The survival rate of CHD patients greatly increased with the quick advancements in surgery and detection approaches. It mainly depends on the kind and severity of CHD, with a survival rate of over 98 percent for milder illnesses [3]. However, these patients continue to have a very high risk of developing lower respiratory infections that cause increased morbidity and mortality [4].

Due to situations of various types of heart abnormalities, CHDs can have a variety of symptoms. A blue tint to the skin (cyanosis), clubbed fingernails, heavy perspiration, intense exhaustion, fatigue, poor feeding, rapid heartbeat, shortness of breath, and chest pain are some of the general symptoms of this illness [5]. Congenital cardiac diseases manifest themselves soon after birth, although symptoms do not appear until early childhood or adolescence. Adulthood is when some complications can arise, such as issues with heart and body growth and development, infections of the sinuses, respiratory tract, throat, lungs, and heart, endocarditis, pulmonary hypertension, high blood pressure, and a heart that cannot pump enough blood, which can result in heart failure [5].

In numerous experimental animal models, cardiac malformations have been created by disrupting specific molecules that act in the developmental pathways involved in myocyte specification, differentiation, or cardiac morphogenesis [1]. The precise genetic, epigenetic, and environmental causes of the many heart abnormalities are still not entirely understood. Researchers have made some progress in their understanding of unusual Mendelian CHD families through human genome analysis and DNA gene sequencing in patient cohorts with CHD throughout the past 40 years of genetic study into heart illnesses.

Although the discovery of disease gene mutations' penetrance is well known, this research has yielded three noteworthy insights: first, human CHD mutations affect a heterogeneous set of molecules that orchestrate cardiac development; second, CHD mutations frequently alter gene-protein dosage; and third identical pathogenic CHD mutations cause a variety of distinct malformations, and this suggests that higher-order interactions are responsible for specific CHD phenotypes.

### 1.2. Types of Congenital Heart Diseases

There are very few misdiagnoses made nowadays since clinical and echocardiographic diagnoses are so precise, but there are still significant diagnostic challenges with the classification and, consequently, with the inclusion as specific lesions. These lesions comprise a range of different types and accordingly varying severities. CHD lesions include isolated ventricular septal defect, patent ductus arteriosus, atrial septal defects of the fossa ovalis (secundum) type, isolated partial anomalous pulmonary venous connection, atrioventricular septal defects, pulmonic stenosis, bicuspid aortic valves, coarctation of the aorta, and mitral incompetence [6].

**Figure 1:**
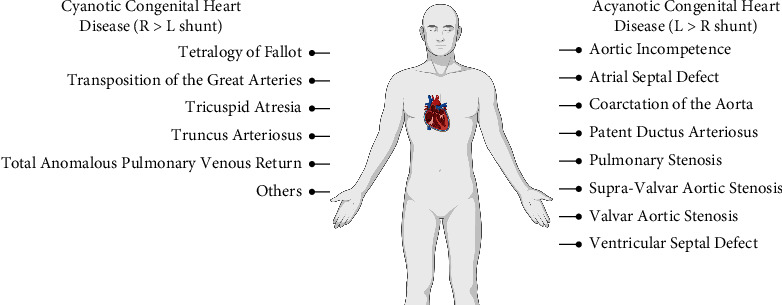
Cyanotic and acyanotic congenital heart diseases. Recreated from [2] via BioRender.

The classification of numerous lesions is inconsistent, and hence the classification of CHD into three types of lesions based on severity is, therefore, highly helpful. The first category presents severe CHD that encompasses cyanotic and acyanotic heart diseases. Most patients who present extremely unwell during the newborn period or early infancy fall into this category (Figure 1). Cyanotic heart disease includes tetralogy of fallot including pulmonary atresia, absent pulmonary valve, and hypoplastic right or left heart. Although right-to-left shunting, insufficient pulmonary blood flow, or common mixing lesions can be used to categorize cyanotic heart lesions, many defects contain several physiologic problems [6]. The “five *T*'s” of cyanotic CHD which include transposition of the great arteries, tetralogy of fallot, truncus arteriosus (also known as “truncus”), total anomalous pulmonary venous connection, and anomalies of the tricuspid valve remain a helpful mnemonic [7]. Acyanotic heart diseases include atrioventricular septal defect, a large ventricular septal defect, and a large patent ductus arteriosus [6]. The second category presents moderate CHD which comprises mild or moderate aortic stenosis or aortic incompetence, pulmonic stenosis or incompetence, and complex forms of ventricular septal defect. The third category presents mild CHD, most patients fall within this category, and this is due to their lack of symptoms, potential lack of substantial murmurs, and frequent early spontaneous resolution of their lesions. This category includes small ventricular septal defect, small patent ductus arteriosus, mild pulmonic stenosis, and others [6].

### 1.3. Noncardiac Cyanosis-Inducing Etiologies

Some patients present as suspects of cyanotic heart defect as their presentation resembles that of Cyanotic heart disease; however, upon cardiological examinations, a heart defect is excluded. Serological markers reveal increased blood methemoglobin (MHb) levels and decreased activity of NADH-dependent MHb reductase which causes methemoglobinemia [8]. Methemoglobinemia, an uncommon yet easily detected condition, may resemble cyanotic CHD. A permanent or temporary enzyme shortage may be caused by toxic substances, particularly nitrate absorption. Methemoglobinemia with subsequent hemoglobin disorders or congenital enzyme deficiencies has a dismal prognosis. The preferred therapeutic and diagnostic tool is methylene blue [9]. As a matter of physiology, hemoglobin loses its capacity to transport molecular oxygen and carbon dioxide when the ferrous (Fe_2_) iron component of hemoglobin is oxidized to the ferric (Fe_3_) state to generate MHb. Cyanosis, compromised aerobic respiration, metabolic acidosis, and in extreme situations, mortality, are all effects of increased MHb levels. To convert MHb back to hemoglobin, erythrocytes contain reduced glutathione, reduced NADPH-MHb reductase, and cytochrome-b5-MHb reductase. When these enzyme systems are overworked, methemoglobinemia becomes fatal [10]. Individuals develop cyanosis as the MHb content rises. The typical symptoms include confusion and tachypnea, followed by anxiety, light-headedness, headache, and tachycardia. Patients may have acidosis, seizures, arrhythmias, and eventually coma and death if the MHb levels rise. For a given MHb concentration, patients with the underlying cardiac, pulmonary, or hematologic disease may have more severe symptoms [11].

## 2. COVID-19/SARS-CoV-2

### 2.1. Overview

The novel severe acute respiratory syndrome coronavirus 2 (SARS-CoV-2) is the cause of the COVID-19 outbreak, also known as COVID-19, which was initially discovered in China in December 2019 [12]. The COVID-19 pandemic was formally classified as a public health emergency of international concern by the World Health Organization in January 2020, as a result of the SARS-CoV-2 virus' quick spread over the world. Coronaviruses belong to the Nidovirales order's Coronaviridae family of enclosed, positive single-stranded RNA viruses with genomes that range in size from 26 to 32 kb [13]. There are currently four identified genera of the virus, namely, alpha (*α*), beta (*β*), gamma (*γ*), and delta (*δ*) [14]. The novel SARS-CoV-2, on the other hand, is a member of the genus coronavirus and has an RNA genome size of 29.9 kb [15]. Research has shown that the SARS-CoV-2 virus is primarily spread between humans by inhalation or contact with droplets that are infectious, with an incubation period of 2 to 14 days [16‐18]. A wide variety of clinical symptoms, ranging from asymptomatic to symptomatic, are associated with SARS-CoV-2 infection, including respiratory symptoms, fever, shortness of breath, cough, dyspnea, viral pneumonia, and in more severe cases, pneumonia, severe acute respiratory syndrome, heart failure, renal failure, and even death [19]. However, respiratory failure, septic shock, renal failure, hemorrhage, and heart failure are the leading causes of death associated with COVID-19.

**Figure 2:**
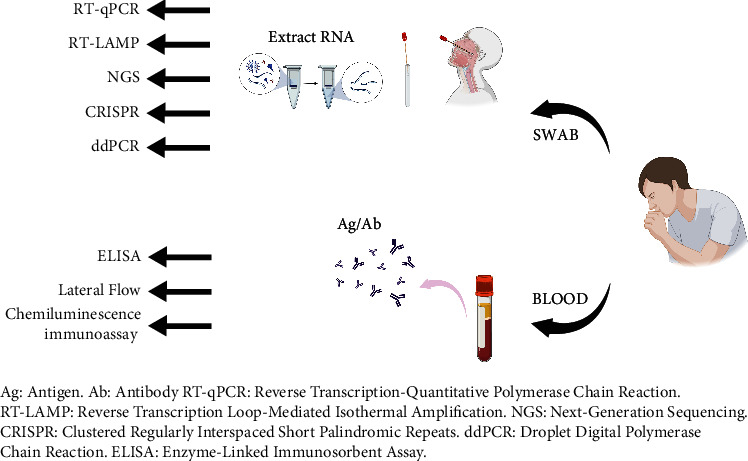
Schematic presentation of analytical methods available for SARS-CoV-2 detection. A sample should be obtained from the patient by means of a long swab that is inserted into the nostrils and is adequately swept against the walls of the nasopharynx. Following sample collection, RNA extraction is performed followed by any of the analytical laboratory methods (RT-qPCR, RT-LAMP, NGS, CRISPR, and ddPCR). Alternatively, a sample can be collected from the patient's serum. Blood is then treated to obtain antigens and/or antibodies present which then can be analyzed via ELISA, lateral flow of chemiluminescence immunoassay. Recreated from [20] via BioRender.

Individual differences in the clinical presentation of new SARS-CoV-2 infection range from asymptomatic presentations to severe respiratory distress syndrome and multiorgan failure. Consequently, it is difficult to make an accurate COVID-19 diagnosis. The epidemiological history, clinical signs, and confirmation by a range of laboratory detection techniques are the main components of the usual clinical diagnosis of COVID-19 (Figure 2) [20].

### 2.2. COVID-19 Pathophysiology

The three phases of COVID-19 represent its pathogenesis. Viral entry into the respiratory epithelium initiates the first phase, which is followed by cellular proliferation. The initial immune response is characterized by moderate symptoms and is characterized by the activation of monocytes and macrophages. Pulmonary vasodilatation and enhanced vascular permeability mark the start of the subsequent phase. Following leukocyte migration, fluid extravasation and pulmonary edema occur. Alveolar injury, hypoxia, heart damage, and stress are thus provoked. The excessive inflammatory response leads to a large cytokine storm in the last phase, which is its defining feature [21].

## 3. COVID-19 in Patients with Cyanotic Congenital Heart Disease

The SARS-CoV-2 infection may directly or indirectly affect the heart by affecting the respiratory system and other organs. In the context of COVID-19, there are three processes that result in cardiac involvement: (1) direct damage brought on by direct viral entry into cardiac cells, (2) hypoxia-induced myocardial ischemia, and (3) an exaggerated, heightened inflammatory response characterized by endothelial overactivation and microvascular thrombi [2, 22]. The effects of this infection on a heart that is pressure- or volume-overloaded in the context of CHD could theoretically be more severe. Furthermore, there are no established risk factors for COVID-19 severity in CHD patients [23]. Patients with CHD frequently exhibit genetic abnormalities, although it is unclear how these syndromes may affect the patient in this case. Physicians should prioritize emergency measures by learning how CHD affects the course and outcome of COVID-19 [23]. Patients with CHD are thought to be at a higher risk of mortality from COVID-19 infection or suffering worse complications. In a study conducted by Bromberg et al., adults with CHD had comparable COVID-19 mortality rates to the general population. While the anatomic complexity does not seem to predict the severity of infection, patients with worse physiological stages are more susceptible, such as cyanosis and pulmonary hypertension [24]. The study yielded a conclusion that male sex, diabetes, cyanosis, pulmonary hypertension, renal insufficiency, and prior hospital admission for heart failure were all risk factors for poor prognosis in COVID-19 and COVID infection-related mortalities [24, 25].

The most typical symptom in these patients is cough, followed by edema, fever, dyspnea, cyanosis, restlessness, and poor feeding in infants. Laboratory results show that some patients exhibit extremely increased C-reactive protein or erythrocyte sedimentation rate. Others develop mild lymphopenia, and some develop thrombocytopenia during their hospital stay. Low oxygen saturation levels also seem to be an alarming laboratory finding [23].

Clinicians are in constant search for an association between CHD patients' contraction of COVID-19 and adverse outcomes of infection. For this sake, some researchers consider hospitalization for COVID-19 requiring noninvasive or invasive ventilation and/or inotropic support, as well as a death outcome to be defining features of a problematic and complicated disease course [26]. Cyanotic lesions, such as unrepaired cyanotic abnormalities or Eisenmenger syndrome, were among the congenital heart anomalies that posed a particularly high risk and are considered the most important predictors of a complicated disease course [26]. Due to a right-to-left shunt as well as severe aberrant pathobiology of the pulmonary tissue and pulmonary vascular bed, individuals with cyanotic heart disease, including those with Eisenmenger syndrome, exhibit persistent hypoxemia and frequently have much lower resting oxygen saturations. In the event of respiratory tract infections with reduced oxygenation, such patients hold a risk of rapidly deteriorating. An increase in right-to-left shunting caused by an increase in pulmonary vascular resistance and an inflammatory-mediated decrease in systemic vascular resistance exacerbates pre-existing hypoxemia in the case of a severe COVID-19 infection [27]. Additionally, the risk of paradoxical embolism is higher for these patients. A worse result in these individuals may also be caused by the potential increased prothrombotic risk brought on by pre-existing hemostatic problems, venous stasis, endothelial damage, and inflammatory response [28]. D-dimer may aid in the early identification of these high risk patients and assist in outcome prediction. Additionally, preliminary findings show that anticoagulant medication appears to be related to decreased mortality in the subgroup satisfying sepsis-induced coagulopathy criteria or with noticeably raised D-dimer levels in patients with severe COVID-19 [29]. The main risk factor is venous stasis, which is frequently visible in the cavopulmonary circuit due to the lack of a pump for both pulmonary blood flow and systemic venous return; therefore, these patients should be given long-term anticoagulation [30]. Even lesser levels of pulmonary involvement can be anticipated to cause the patients status to worsen. To avoid stroke from an air embolism, air filters should be installed on all venous cannulas in patients with (residual) right-to-left shunts [31].

**Figure 3:**
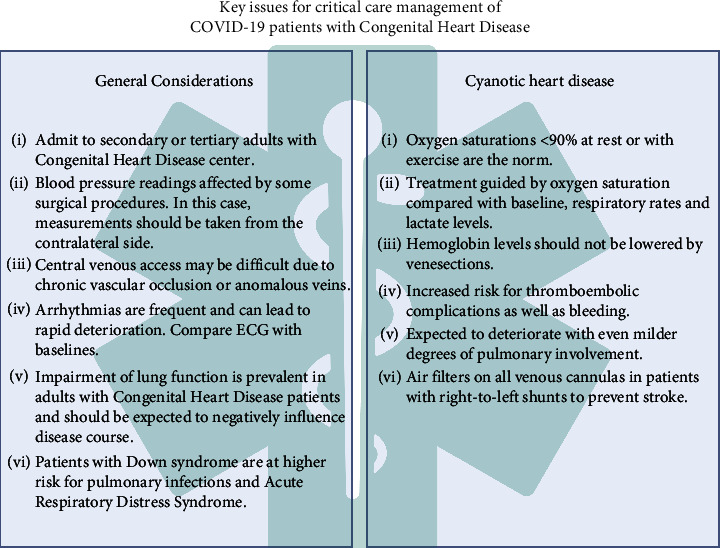
Summary of specific considerations for severely affected adults with CHD patients and those with cyanotic heart disease. Recreated from [31] via BioRender.

Oxygen saturation levels of less than 90% at rest or during activity may be typical in adults with CHD patients with cyanotic heart disease. Cyanosis can be remarkable when the fingers and toes are clubbed. In addition to the present measurements, treatment decisions must be based on the pre-COVID-19 baseline oxygen saturation [31]. Instead of absolute values of oxygen saturation, thresholds for oxygen dosing or switching to mechanical ventilatory assistance must be based on variables such as respiratory rate and lactate levels. Venesections should not be performed since chronic cyanosis causes an adaptive increase in hemoglobin levels that are needed in this situation (Figure 3) [31].

### 3.1. Impact of COVID-19 on Pediatric Patients with Congenital Heart Disease

According to recent advances, the COVID-19 infection and the pandemic's collateral damage present a burden on pediatric patients with CHD. Most infected pediatrics have mild to moderate illnesses, and laboratory and radiographic data show significant interindividual variation. However, cardiac involvement in children with COVID-19 who are healthy has been observed and is related to a number of factors [2]. Children can develop myocarditis, arrhythmias, cardiogenic shock, and catastrophic multisystem inflammatory syndrome. Children who have been infected have reported cases of asymptomatic, mild, moderate, severe, and critically sick cases. Patients with CHD, especially those with cyanotic abnormalities, are more likely to need intensive care unit (ICU) hospitalization and artificial respiratory assistance. COVID-19 may aggravate hypoxemia and impair tissue perfusion in these patients. Additionally, patients with complex CHD who also have pulmonary hypertension, immunodeficiencies (such as DiGeorge syndrome), and other concomitant diseases such as reduced myocardial contractility are at risk of developing severe and critical COVID-19 illness [2].

### 3.2. Vaccination for COVID-19 in Congenital Heart Disease Patients

Until today, there are not enough data on the COVID-19 vaccine's acceptability, immunogenicity, and safety in adults with CHD. In a study conducted by Fusco et al. on COVID-19 vaccination in adults with CHD has revealed that COVID-19 vaccinations, had it been Pfizer-BioNTech BNT162b2 vaccine, Moderna, or AstraZeneca-ChAdOx1, have acceptable immunogenicity and seem safe in adults with CHD. However, the most susceptible patients in the study displayed a reduced antibody response. Patients in this study reported symptoms after the first and second doses. Symptoms duration was always limited, there were no allergic responses, and the most frequent symptoms were headaches, fever, muscle soreness, gastrointestinal disturbances, exhaustion, and dizziness [32]. To this end, studies provided comforting information about the vaccinations' good safety profile, with the majority of side effects being brief and mild, similar to what has previously been documented in the general population worldwide [33]. It appears that vaccination avoidance based on worries about vulnerability due to the underlying heart disease is not justified. Vaccine administration is a low-risk action. As noted before, regardless of prior viral infection, adults with CHD patients with advanced physiological stages may have reduced antibody responses; however, this does not diminish the positive effect of these patients receiving COVID-19 vaccines [32].

### 3.3. Specific Considerations for Severely Affected Adults with Congenital Heart Disease Patients

When handling severely impacted adults with CHD patients with complicated underlying lesions, a detailed study of the underlying anatomy and pathophysiology is necessary, and they should be admitted to secondary or tertiary adults with CHD centers. Many adults with CHD patients are prone to developing arrhythmias, which frequently require immediate management to avoid decompensation [34]. Some concerns are relevant for adults with CHD patients who are hospitalized in critical care units. For instance, common prior surgical procedures (Blalock–Taussig–Thomas shunts and subclavian flap) can alter blood pressure readings, so measures should be performed from the contralateral side. Chronic arterial occlusion typically prevents central venous access because of prior critical care stays, numerous operations, and pacemaker leads. Large catheters, such as those used for hemofiltration, may have trouble fitting through a persistently tiny right superior vena cava due to a persistently small right superior vena cava.

Patients with Down syndrome are more likely to develop lung infections and acute respiratory distress syndrome which are frequently linked to CHD and immunological abnormalities (Figure 3) [35].

In a study conducted by Sachdeva et al. on the outcome of COVID-19-positive children with heart disease and grown-ups with CHD, researchers aimed at identifying risk factors that might be associated with mortality in those patients. A total of 94 patients were included, and they were presented with either symptomatic or asymptomatic COVID-19 infection. In this study, researchers classified the types of heart diseases into obstructive, acyanotic, and cyanotic CHD and acquired heart disease in children. 31 patients had acyanotic CHD, and 39 patients were cyanotic, with >80% of the patients being unoperated. Based on the occurrence of cyanotic episodes, refractory heart failure, persistent shock, or the need for ventilatory support, the degree of sickness upon presentation was divided into severe and nonsevere illness [36]. Children with CHD are known to have poorer prognosis with common respiratory viral and bacterial infections, and pneumonia are the most prevalent noncardiac cause of mortality in these children [36, 37]. Furthermore, COVID-19 pneumonia in a child with CHD can result in hypercapnic vasoconstriction, worsening *V*/*Q* mismatch, embolic events, worsening pulmonary hypertension, and progressive hypoxia. According to this study, children with cyanotic CHD are probably more prone to experience this combined impact, which will decrease tissue oxygenation and perfusion, hence, making these patients face a fatal battle against COVID-19 infection. [38].

Literature has some studies that contradict the former findings. Some studies concluded that children and young adults with an underlying cardiac condition rarely had to be hospitalized for coronavirus illness. Cardiovascular risk factors were not linked to an increased chance of hospitalization; however, extracardiac comorbidities were linked. The severe acute respiratory syndrome of coronavirus did not seem to be associated with the traditional cardiac risk factors for more severe acute respiratory infections in children. For instance, in these studies, most patients who required treatment had ventricular dysfunction, or had persistent, substantial cardiac abnormalities that affected hemodynamics, reported having mild or asymptomatic infections [39]. The patients with palliated single ventricle CHD and those with residual cyanotic CHD who are among the people most at risk for developing severe respiratory infections did not require hospitalization for the severe acute respiratory syndrome of coronavirus infection during the study period. When comorbidities were examined individually as risk factors, only chronic lung disease and immune suppression showed statistically significant correlations with hospitalization. This conclusion may be partially explained by the limited sample size of these studies, which reduces the sensitivity to identifying possible risk factors in cardiac patients [39].

To this end, according to the anatomical and physiological stage classification, patients with complex CHD, such as Fontan patients, cyanotic congenital heart defects (unrepaired or palliated), single ventricles, and pulmonary atresia, should be regarded as having high risk of complications from COVID-19 infection because of a decreased functional reserve. When patients are admitted, the treatment plan should address indicators of end-organ damage, symptomatic support, and management of respiratory failure. Patients with mild illness can be handled with noninvasive measures of additional oxygen support [40]. However, patients with severe COVID-19 disease frequently require intubation to improve oxygenation and ventilation because they have symptoms resembling acute respiratory distress syndrome. This can be difficult for Fontan patients because increased intrathoracic pressure brought on by positive pressure ventilation has detrimental effects on intrapulmonary and intracardiac hemodynamics, resulting in lower preload and, ultimately, decreased systemic cardiac output. Positive end expiratory pressure (PEEP) should be kept within the range to maintain the lung's functional residual capacity and prevent atelectasis and hypoxia-related vasoconstriction if intubation is necessary [40].

### 3.4. Hospitalization of Congenital Heart Disease Patients with COVID-19

When looking at the hospitalization of these patients, and according to recent research by the American Heart Association, people who were hospitalized with COVID-19 infection and had a congenital heart defect were more likely to experience serious sickness along with a complicated disease course or die than those who did not have a congenital heart problem. Individuals with congenital cardiac defects who contracted COVID-19 were also more likely to need ventilator support or treatment in an ICU [41]. Patients who had a heart defect and other medical issues were older than 50, or were male were among those who were most at risk for developing the most severe COVID-19 sickness [42]. In a study conducted by Diaz et al., the researchers attempted to describe patient characteristics in those with and without CHDs during hospitalization for COVID-19. At the time of the COVID-19 hospitalization, the results of the study revealed that patients with CHDs were considerably more likely to have obesity, acute pulmonary hypertension, venous thromboembolism, acute ischemic stroke, acute arrhythmia, myocardial damage, and heart failure but not respiratory failure. Patients with CHDs were much more likely to be admitted to the ICU and had a significantly longer median length of stay than patients without CHDs [43]. Furthermore, it is important to identify the risk factors associated with the increased mortality rates in these hospitalized patients. Studies had implied that physical symptoms such as anorexia, nausea, vomiting, diarrhea, chest discomfort, myalgia, and fever have no bearing on a patient's mortality. However, the mortality rates of COVID-19 cardiovascular patients were significantly correlated to symptoms such as headache, loss of consciousness, oxygen saturation below 93%, and the requirement for mechanical ventilation [44].

## 4. Materials and Methods

We performed a comprehensive and updated search on the severity of SARS-CoV-2 infection in patients with cyanotic CHD. Our search included some studies and reports published in the literature. Our search relied mostly on PubMed, Medline, and Google Scholars. The keywords used for the search included congenital heart disease, cyanosis, COVID-19, and SARS-CoV-2. Upon our search, studies handling the association between COVID-19 and CHD were retrieved, and their conclusions were compiled in our review. The studies are tabulated (Table 1), which shows the objectives, methodologies, and results of each study. Additionally, many narrative reviews were also retrieved to complement and support the information presented in our review.

## 5. Discussion

According to the literature presented above, the comorbidities and the complexity of the heart defects were thought to be the main risk factors for poor outcomes in the case of COVID-19. Patients with cyanotic heart disease are at a particularly high risk when challenged with the infection. Upon infection with COVID-19, the existing cyanotic CHD manifests itself dramatically. Patients with cyanotic heart disease exhibit continuous hypoxemia and frequently have considerably lower resting oxygen saturations because of a right-to-left shunt as well as severe abnormal pathobiology of the pulmonary tissue [27]. These patients are at an increased risk for rapid health deterioration in the setting of respiratory tract infections with inadequate oxygenation [27]. Importantly, patients have a higher risk of paradoxical embolism [28]. This risk stems from the concomitant prothrombotic risk due to pre-existing hemostasis issues, venous stasis, endothelial damage, and inflammatory response may also result in a worse outcome [28]. Despite all these findings, a study conducted by Sabatino et al. aimed to evaluate the clinical traits and prognoses of COVID-19-affected CHD patients [45]. The cohort study showed that patients with CHD experienced a mild COVID-19 clinical course in contrary to the high case-fatality rates observed in earlier studies on patients with cardiovascular comorbidities [45]. However, this study had several limitations but was reassuring and comforting for CHD patients [45].

Along the course of COVID-19 pandemic, the approach to the vulnerable patients given their different presentations was problematic. Professionals in charge of particularly high risk groups, such as adult patients with CHD, were forced to make difficult choices during the initial onset of the COVID-19 pandemic [46]. During the early weeks of the pandemic, pre-existing cardiovascular illnesses were discovered to be a key indicator of a poor prognosis in cases of infection with the novel SARS-CoV-2 [46]. It was not known for a long time whether this link held true for the predominantly adult individuals with CHD. A prospective multicenter European registry published in March 2023 attempted to assess the changes in risk stratification of adults with CHD patients [46]. By contrasting the results of two surveys given to experts in the field of adults with CHD at two different points during the pandemic, at the start and soon after the first outcome data on adults with CHD patients with COVID-19 were available, they determined changes in risk stratification of adults with CHD patients during the pandemic [46]. The overall risk perception was lower in the second survey than it was in the first when assessing the significance of general and adults with CHD-specific risk factors for a complicated disease course in the case of COVID-19 among the patients [46]. This was true even for risk factors related to physiological stage, which have been linked to poor prognostic outcomes in adults with CHD patients with COVID-19 [24, 46].

This implies that the same risk factors that suggest poor outcomes in COVID-19 cases as are seen in the general population similarly influence the outcomes of adults with CHD patients [46]. Although COVID-19 individuals with cyanotic heart illnesses were at risk for a worst outcome in general, the anatomical complexity of CHD per se did not appear to be related to the increased mortalities and morbidities in case of COVID-19 infection [46]. The prognosis of patients in later waves of the pandemic was improved by the knowledge gathered during the first wave, and hence, approach to the patients has changed significantly along to pandemic [46].

## 6. Conclusion

The COVID-19 pandemic has acutely affected patients with the underlying medical conditions. However, the effects of this infection in the context of CHD could conceivably be more severe, particularly in patients with cyanotic CHD. Based on the anatomical and physiological stage classification of the cardiac status, physicians should have specific considerations when handling a cyanotic patient. However, more studies need to address the clinical presentation of cyanotic CHD patients in particular and investigate the changes of this presentation along the various waves of COVID-19 pandemic. This would allow for a better healthcare provision and better treatment outcomes.

## Figures and Tables

**Table 1:** Characteristics of included studies in our review.

Authors	Date	Title	Study design	Objective	Methods	Results
Moons et al.	2010	Temporal trends in survival to adulthood among patients born with congenital heart disease from 1970 to 1992 in Belgium	Retrospective study	To investigate the proportion of CHD patients born between 1990 and 1992 who survived into adulthood and to compare their survival with that of CHD patients born in earlier eras and evaluate survival as a function of the type of heart defect	Authors reviewed the CHD program administrative and clinical database at the university hospitals Leuven (Leuven, Belgium) and analyzed the records of 7497 CHD patients born from 1970 to 1992	This study demonstrated that almost 90% of children with CHD have the prospect of surviving into adulthood

Huang et al.	2020	Clinical features of patients infected with 2019 novel coronavirus in Wuhan, China	Prospective study	To report the epidemiological, clinical, laboratory, and radiological characteristics and treatment and clinical outcomes of COVID-19 patients	All patients with suspected 2019-nCoV were admitted to a designated hospital in Wuhan. Prospectively collected and analyzed data on patients with laboratory-confirmed 2019-nCoV infection by real-time RT-PCR and next-generation sequencing. Data were obtained with standardised data collection forms shared by WHO and the International Severe Acute Respiratory and Emerging Infection Consortium from electronic medical records. Researchers also directly communicated with patients or their families to ascertain epidemiological and symptom data. Outcomes were also compared between patients who had been admitted to the ICU and those who had not	The 2019-nCoV infection caused clusters of severe respiratory illness similar to severe acute respiratory syndrome coronavirus and was associated with ICU admission and high mortality

Broberg et al.	2021	COVID-19 in adults with congenital heart disease	Cohort study	This study sought to define the impact of COVID-19 in adults with CHD and to identify risk factors associated with adverse outcomes	Adults (age 18 years or older) with CHD and with confirmed or clinically suspected COVID-19 were included from CHD centers worldwide. Data collection included anatomic diagnosis and subsequent interventions, comorbidities, medications, echocardiographic findings, presenting symptoms, course of illness, and outcomes. Predictors of death or severe infection were determined	COVID-19 mortality in adults with CHD is commensurate with the general population. The most vulnerable patients are those with worse physiological stage, such as cyanosis and pulmonary hypertension, whereas anatomic complexity does not appear to predict infection severity

Ruperti-Repilado et al.	2021	Risk stratification of adults with congenital heart disease during the COVID-19 pandemic: insights from a multinational survey among European experts	Cross-sectional study	The study aimed to provide an expert view on risk stratification while awaiting results from observational studies	This study was an initiative of the EPOCH (European Collaboration for Prospective Outcome Research in Congenital Heart) disease. Among nine European countries (Austria, Belgium, Denmark, France, Germany, Italy, the Netherlands, Spain, and Switzerland), 24 experts from 23 tertiary adults with CHD centers participated in the survey. Adults with CHD experts were asked to identify adults with CHD-specific COVID-19 risk factors from a list of potential outcome predictors and to estimate the risk of adverse COVID-19 outcomes in seven commonly seen patient scenarios	Pulmonary arterial hypertension, Fontan palliation, and cyanotic heart disease were widely considered as risk factors for poor outcome in COVID-19. However, there was a marked disparity in risk estimation for other clinical scenarios. We are in urgent need of outcome studies in adults with CHD suffering from COVID-19

Schwerzmann et al.	2021	Clinical outcome of COVID-19 in patients with adult congenital heart disease	Cohort study	The aim of this study was to collect clinical outcome data and to identify risk factors for a complicated course of COVID-19 in adults with CHD	Twenty-five adults with CHD centers in nine European countries participated in the study. Consecutive patients with CHD diagnosed with COVID-19 presenting to one of the participating centers between 27 March and 6 June 2020 were included. A complicated disease course was defined as hospitalization for COVID-19 requiring noninvasive or invasive ventilation and/or inotropic support, or a fatal outcome	Among patients with CHD, general risk factors (age, obesity and multiple comorbidities) are associated with an increased risk of complicated COVID-19 course. Congenital cardiac defects at particularly high risk were cyanotic lesions, including unrepaired cyanotic defects or Eisenmenger syndrome

Sathananthan et al.	2019	Clinical importance of Fontan circuit thrombus in the adult population: significant association with increased risk of cardiovascular events	Retrospective study	The objectives of this study were to determine (1) the incidence of Fontan circuit thrombus and proportion of silent thrombus; (2) any association between Fontan circuit thrombus and markers of Fontan circulatory dysfunction; and (3) the association of Fontan circuit thrombus with adverse cardiac outcomes	The group conducted a retrospective review of adult patients who underwent the Fontan procedure (aged >18 years) followed at St. Paul's hospital who underwent cardiac computed tomography or magnetic resonance imaging assessment (*n* = 67). Fontan circulatory dysfunction markers included clinical heart failure, *N*-terminal probrain natriuretic peptide, ventricular dysfunction, atrioventricular valvular regurgitation, refractory arrhythmias, declining exercise capacity, and hepatic/renal dysfunction. Adverse cardiac outcomes were death, heart transplantation, or surgery for Fontan revision or atrioventricular valve replacement	Given the incidence of Fontan circuit thrombus and association with adverse cardiac outcomes, routine surveillance of the Fontan circuit should strongly be considered. The identification of thrombus should lead to anticoagulation implementation/optimization, along with screening/intervention for reversible Fontan circulatory issues in an attempt to prevent adverse cardiac outcomes

Fusco et al.	2021	COVID-19 vaccination in adults with congenital heart disease: real-world data from an Italian tertiary center	Retrospective study	To assess COVID-19 vaccine safety, immunogenicity and acceptance in adults with congenital heart disease	Adults with CHD patients who were offered COVID-19 vaccination from January to June 2021 were included. Data on adverse events, on patients' attitude towards vaccination and antispike IgG titre were retrospectively collected. A group of healthy individuals with similar age and sex undergoing vaccination was included for comparison	COVID-19 vaccines appear safe in adults with CHD with satisfactory immunogenicity. However, the most vulnerable patients showed lower antibody response. Adults with CHD team may play a key role in vaccine acceptance

Yuan et al.	2020	Safety, tolerability, and immunogenicity of COVID-19 vaccines: a systematic review and meta-analysis	Systematic review and meta analysis	The paper aimed to summarize reliable medical evidence by the meta-analysis of all published clinical trials that investigated the safety, tolerability, and immunogenicity of vaccine candidates against COVID-19, caused by SARS-CoV-2	The PubMed, cochrane library, EMBASE, and medRxiv databases were used to select the studies. 7094 articles were identified initially and 43 were retrieved for more detailed evaluation. 5 randomized, double-blind, placebo-controlled trials were selected. A total of 1604 subjects with either vaccines or placebo infections were included in the meta-analysis within the scope of these articles	Current COVID-19 vaccine candidates are safe, tolerated, and immunogenic, which provides important information for further development, evaluation, and clinical application of COVID-19 vaccine

Sachdeva et al.	2021	Effect of acute lower respiratory tract infection on pulmonary artery pressure in children with post-tricuspid left-to-right shunt	Prospective observational study	To examine the influence of clinically severe lower respiratory tract infection on pulmonary artery pressure in children having CHD with post-tricuspid left-to-right shunt, as it may have physiological and clinical implications	45 children with post-tricuspid left-to-right shunt and clinically severe lower respiratory tract infection were evaluated during the illness and 2 weeks after its resolution. Pulmonary artery systolic pressure was estimated noninvasively using shunt gradient by echocardiography and systolic blood pressure measured noninvasively	In the absence of hypoxia or acidosis, lower respiratory tract infection in patients with post-tricuspid left-to-right shunt causes only a mild increase in the pulmonary artery systolic pressure that is statistically significant, but may not be clinically significant in the majority of patients

Medrano et al.	2007	Respiratory infection in congenital cardiac disease. Hospitalizations in young children in Spain during 2004 and 2005: the CIVIC epidemiologic study	Cohort study	To evaluate the rate of hospitalization for acute respiratory tract infection in children less than 24 months with hemodynamically significant congenital cardiac disease, and to describe associated risk factors, preventive measures, etiology, and clinical course	760 subjects were followed from October 2004 through April 2005 in an epidemiological, multicentric, observational, follow-up, prospective study involving 53 Spanish hospitals	Hospital admissions for respiratory infection in young children with hemodynamically significant congenital cardiac disease are mainly associated with noncardiac conditions, which may be genetic, malnutrition, or respiratory, and to cardiopulmonary bypass. Respiratory syncytial virus was the most commonly identified infectious agent. Incomplete immunoprophylaxis against the virus increased the risk of hospitalization

Sachdeva et al.	2021	Outcome of COVID-19-positive children with heart disease and grown-ups with congenital heart disease: a multicentric study from India	Retrospective, multicentric, observational study	To analyze outcome data and identify risk factors associated with mortality in children with heart disease and grown-ups with CHD (GUCH) who had a laboratory-confirmed COVID-19 infection	The study included children with heart disease and GUCH population, who presented with either symptomatic or asymptomatic COVID-19 infection to any of the participating centers. COVID-19-negative patients admitted to these centers constituted the control group	Children with heart disease are at a higher risk of death when they acquire COVID-19 infection. Systematic preventive measures and management strategies are needed for improving the outcomes

Fisher et al.	2021	Characteristics and outcomes of acute COVID-19 infection in paediatric and young adult patients with underlying cardiac disease	Retrospective study	To describe outcomes of acute COVID-19 in paediatric and young adult patients with underlying cardiac disease and evaluate the association between cardiac risk factors and hospitalization	A retrospective single-institution review of patients with known cardiac disease and positive severe acute respiratory syndrome coronavirus 2 RT-PCR from 1 March, 2020 to 30 November, 2020. Extracardiac comorbidities and cardiac risk factors were compared between those admitted for COVID-19 illness and the rest of the cohort using univariate analysis	Hospitalizations for COVID-19 were rare among children and young adults with underlying cardiac disease. Extracardiac comorbidities like pulmonary disease were associated with the increased risk of hospitalization while cardiac risk factors were not

Lewis et al.	2020	Impact of coronavirus disease 2019 (COVID-19) on patients with congenital heart disease across the lifespan: the experience of an academic congenital heart disease center in New York city	Retrospective study	To assess the impact and predictors of COVID-19 infection and severity in a cohort of patients with CHD at a large CHD center in New York city	A retrospective review of all individuals with CHD followed at Columbia University Irving Medical Center who were diagnosed with COVID-19 between March 1, 2020 and July 1, 2020. The primary end point was moderate/severe response to COVID-19 infection defined as (1) death during COVID-19 infection; or (2) need for hospitalization and/or respiratory support secondary to COVID-19 infection	The number of symptomatic patients with COVID-19 was relatively low. Patients with CHD with a genetic syndrome and adults at advanced physiological stage were at highest risk for moderate/severe infection

Diaz et al.	2022	Describing characteristics of adults with and without congenital heart defects hospitalized with COVID-19	Retrospective study	To describe patient characteristics in adults with and without congenital heart defects during hospitalization for COVID‐19	The team analyzed data collected by optum®, a nationally representative database of electronic medical records, for 369 adults with CHDs and 41,578 without CHDs hospitalized for COVID‐19 between January 1, 2020, and December 10, 2020. We used poisson regression to describe and compare epidemiologic characteristics, heart‐related conditions, and severe outcomes between these two groups	Adults with CHD appear to be at greater risk for more severe CHD, including greater risk of ICU admission and longer length of hospital stays

Alizadehsani et al.	2022	Factors associated with mortality in hospitalized cardiovascular disease patients infected with COVID-19	Retrospective observational study	To determine risk factors, symptoms, and comorbidities leading to mortality in CVD patients who are hospitalized with COVID-19	This study was conducted on 660 hospitalized patients with CVD and COVID-19 recruited between january 2020 and january 2021 in Iran. All patients were diagnosed with the previous history of CVD such as angina, myocardial infarction, heart failure, cardiomyopathy, abnormal heart rhythms, and CHD before they were hospitalized for COVID-19. The team collected data on patient's signs and symptoms, clinical and paraclinical examinations, and any underlying comorbidities. *t*-test was used to determine the significant difference between the two deceased and alive groups. In addition, the relation between pairs of symptoms and pairs of comorbidities has been determined via correlation computation	Signs and symptoms such as fever, cough, myalgia, chest pain, chills, abdominal pain, nausea, vomiting, diarrhea, and anorexia had no impact on patients' mortality. There was a significant correlation between COVID-19 cardiovascular patients' mortality rate and symptoms such as headache, loss of consciousness, oxygen saturation less than 93%, and need for mechanical ventilation

Sabatino et al.	2020	COVID-19 and congenital heart disease: results from a nationwide survey	Multicentric observational nationwide survey	To assess clinical characteristics and outcomes in patients with CHD affected by COVID-19	A nationwide survey aimed at evaluating consecutive patients with CHD admitted to Italian CHD units affiliated and associated with the CHD working group of the Italian Society of Cardiology, during a six-week period of the initial COVID-19 outbreak in Italy: 21 February–4 April. All patients admitted with CHD, who were diagnosed with COVID-19 and either treated and discharged or who died during hospitalization in the 6-week window, were included independently of their age	Despite previous reports pointing to a higher case-fatality rate among patients with cardiovascular comorbidities, this study observed a mild COVID-19 clinical course in our cohort of CHD patients

Ruperti-Repilado et al.	2023	The Coronavirus disease pandemic among adult congenital heart disease patients and the lessons learnt—results of a prospective multicenter European registry	Prospective study	To assess changes in risk stratification and outcomes of adults with CHD patients suffering from COVID-19 between March 2020 and April 2021	638 patients (*n* = 168 during the first wave and *n* = 470 during the subsequent waves) were included (median age 34 years, 52% women). Main independent predictors for a complicated disease course were male sex, increasing age, a BMI >25 kg/m^2^, having ≥2 comorbidities, suffering from a cyanotic heart disease or having suffered COVID-19 in the first wave vs. subsequent waves	Apart from cyanotic heart disease, general risk factors for poor outcome in case of COVID-19 reported in the general population are equally important among adults with CHD patients. Risk perception among CHD experts decreased during the course of the pandemic

## References

[1] Fahed A. C., Gelb B. D., Seidman J. G., Seidman C. E. (2013). Genetics of congenital heart disease: the glass half empty. *Circulation Research*.

[2] Zareef R. O., Younis N. K., Bitar F., Eid A. H., Arabi M. (2020). COVID-19 in pediatric patients: a focus on CHD patients. *Frontiers in Cardiovascular Medicine*.

[3] Moons P., Bovijn L., Budts W., Belmans A., Gewillig M. (2010). Temporal trends in survival to adulthood among patients born with congenital heart disease from 1970 to 1992 in Belgium. *Circulation*.

[4] Geskey J. M., Cyran S. E. (2012). Managing the morbidity associated with respiratory viral infections in children with congenital heart disease. *International Journal of Pediatrics*.

[5] Sun R., Liu M., Lu L., Zheng Y., Zhang P. (2015). Congenital heart disease: causes, diagnosis, symptoms, and treatments. *Cell Biochemistry and Biophysics*.

[6] Hoffman J. I. E., Kaplan S. (2002). The incidence of congenital heart disease. *Journal of the American College of Cardiology*.

[7] Waldman J. D., Wernly J. A. (1999). Cyanotic congenital heart disease with decreased pulmonary blood flow in children. *Pediatric Clinics of North America*.

[8] Jabłońska-Skwiecińska E., Wierzbicka M., Kubicka K. (1989). [Cyanosis in children caused by inherited methemoglobinemia due to deficiency of NADH-dependent methemoglobin reductase in erythrocytes]. *Pediatria Polska*.

[9] Pangaud N., Sassolas F., Bozio A. (1991). Non-cardiac cyanosis: methemoglobinemia in infants. *Pediatrie*.

[10] Anderson C. M., Woodside K. J., Spencer T. A., Hunter G. C. (2004). Methemoglobinemia: an unusual cause of postoperative cyanosis. *Journal of Vascular Surgery*.

[11] Wright R. O., Lewander W. J., Woolf A. D. (1999). Methemoglobinemia: etiology, pharmacology, and clinical management. *Annals of Emergency Medicine*.

[12] Wang C., Horby P. W., Hayden F. G., Gao G. F. (2020). A novel coronavirus outbreak of global health concern. *The Lancet*.

[13] Su S., Wong G., Shi W. (2016). Epidemiology, genetic recombination, and pathogenesis of coronaviruses. *Trends in Microbiology*.

[14] Perlman S., Netland J. (2009). Coronaviruses post-SARS: update on replication and pathogenesis. *Nature Reviews Microbiology*.

[15] Wu F., Zhao S., Yu B. (2020). Author Correction: a new coronavirus associated with human respiratory disease in China. *Nature*.

[16] Lin Q., Zhao S., Gao D. (2020). A conceptual model for the coronavirus disease 2019 (COVID-19) outbreak in Wuhan, China with individual reaction and governmental action. *International Journal of Infectious Diseases*.

[17] Liu Y., Gayle A. A., Wilder-Smith A., Rocklöv J. (2020). The reproductive number of COVID-19 is higher compared to SARS coronavirus. *Journal of Travel Medicine*.

[18] Rohit A., Rajasekaran S., Karunasagar I., Karunasagar I. (2020). Fate of respiratory droplets in tropical vs. temperate environments and implications for SARS-CoV-2 transmission. *Medical Hypotheses*.

[19] Huang C., Wang Y., Li X. (2020). Clinical features of patients infected with 2019 novel coronavirus in Wuhan, China. *The Lancet*.

[20] Rai P., Kumar B. K., Deekshit V. K., Karunasagar I., Karunasagar I. (2021). Detection technologies and recent developments in the diagnosis of COVID-19 infection. *Applied Microbiology and Biotechnology*.

[21] Abi Nassif T., Fakhri G., Younis N. K. (2021). Cardiac manifestations in COVID-19 patients: a focus on the pediatric population. *The Canadian Journal of Infectious Diseases & Medical Microbiology*.

[22] Giustino G., Pinney S. P., Lala A. (2020). Coronavirus and cardiovascular disease, myocardial injury, and arrhythmia: JACC focus seminar. *Journal of the American College of Cardiology*.

[23] Esmaeeli H., Ghaderian M., Zanjani K. S., Ghalibafan S. F., Mahdizadeh M., Aelami M. H. (2021). COVID-19 in children with congenital heart diseases: a multicenter case series from Iran. *Case Reports in Pediatrics*.

[24] Broberg C. S., Kovacs A. H., Sadeghi S. (2021). COVID-19 in adults with congenital heart disease. *Journal of the American College of Cardiology*.

[25] Ruperti-Repilado F. J., Tobler D., Greutmann M. (2021). Risk stratification of adults with congenital heart disease during the COVID-19 pandemic: insights from a multinational survey among European experts. *Open Heart*.

[26] Schwerzmann M., Ruperti-Repilado F. J., Baumgartner H. (2021). Clinical outcome of COVID-19 in patients with adult congenital heart disease. *Heart*.

[27] Creel-Bulos C., Hockstein M., Amin N., Melhem S., Truong A., Sharifpour M. (2020). Acute cor pulmonale in critically ill patients with Covid-19. *New England Journal of Medicine*.

[28] Bikdeli B., Madhavan M. V., Jimenez D. (2020). COVID-19 and thrombotic or thromboembolic disease: implications for prevention, antithrombotic therapy, and follow-up. *Journal of the American College of Cardiology*.

[29] Kollias A., Kyriakoulis K. G., Dimakakos E., Poulakou G., Stergiou G. S., Syrigos K. (2020). Thromboembolic risk and anticoagulant therapy in COVID-19 patients: emerging evidence and call for action. *British Journal of Haematology*.

[30] Sathananthan G., Johal N., Verma T. (2019). Clinical importance of fontan circuit thrombus in the adult population: significant association with increased risk of cardiovascular events. *Canadian Journal of Cardiology*.

[31] Radke R. M., Frenzel T., Baumgartner H., Diller G. P. (2020). Adult congenital heart disease and the COVID-19 pandemic. *Heart*.

[32] Fusco F., Scognamiglio G., Merola A. (2021). COVID-19 vaccination in adults with congenital heart disease: real-world data from an Italian tertiary centre. *International Journal of Cardiology Congenital Heart Disease*.

[33] Yuan P. (2020). *Safety, tolerability, and immunogenicity of COVID-19 vaccines: a systematic review and meta-analysis*.

[34] Hernández-Madrid A., Paul T., Abrams D. (2018). Arrhythmias in congenital heart disease: a position paper of the European heart rhythm association (EHRA), association for European paediatric and congenital cardiology (AEPC), and the European society of cardiology (ESC) working group on grown-up congenital heart disease, endorsed by HRS, PACES, APHRS, and SOLAECE. *EP Europace*.

[35] Colvin K. L., Yeager M. E. (2017). What people with Down Syndrome can teach us about cardiopulmonary disease. *European Respiratory Review*.

[36] Sachdeva S., Kothari S. S., Gupta S. K., Ramakrishnan S., Saxena A. (2021). Effect of acute lower respiratory tract infection on pulmonary artery pressure in children with post-tricuspid left-to-right shunt. *Cardiology in the Young*.

[37] Medrano C., Garcia-Guereta L., Grueso J. (2007). Respiratory infection in congenital cardiac disease. Hospitalizations in young children in Spain during 2004 and 2005: the CIVIC Epidemiologic Study. *Cardiology in the Young*.

[38] Sachdeva S., Ramakrishnan S., Choubey M. (2021). Outcome of COVID-19-positive children with heart disease and grown-ups with congenital heart disease: a multicentric study from India. *Annals of Pediatric Cardiology*.

[39] Fisher J. M., Badran S., Li J. T., Votava-Smith J. K., Sullivan P. M. (2021). Characteristics and outcomes of acute COVID-19 infection in paediatric and young adult patients with underlying cardiac disease. *Cardiology in the Young*.

[40] Ahluwalia N., Love B., Chan A., Zaidi A. N. (2020). COVID-19 in an adult with tricuspid Atresia S/P fontan palliation. *Journal of the American College of Cardiology: Case Reports*.

[41] Lewis M. J. (2020). Impact of coronavirus disease 2019 (COVID‐19) on patients with congenital heart disease across the lifespan: the experience of an academic congenital heart disease center in New York city. *Journal of the American Heart Association*.

[42] Downing K. F., Simeone R. M., Oster M. E., Farr S. L. (2022). Critical illness among patients hospitalized with acute COVID-19 with and without congenital heart defects. *Circulation*.

[43] Diaz P., Coughlin W., Lam W. (2022). Describing characteristics of adults with and without congenital heart defects hospitalized with COVID-19. *Birth Defects Research*.

[44] Alizadehsani R., Eskandarian R., Behjati M. (2022). Factors associated with mortality in hospitalized cardiovascular disease patients infected with COVID-19. *Immunity, Inflammation and Disease*.

[45] Sabatino J., Ferrero P., Chessa M. (2020). COVID-19 and congenital heart disease: results from a nationwide survey. *Journal of Clinical Medicine*.

[46] Ruperti-Repilado F. J., Baumgartner H., Bouma B. (2023). The coronavirus disease pandemic among adult congenital heart disease patients and the lessons learnt - results of a prospective multicenter european registry. *International Journal of Cardiology Congenital Heart Disease*.

